# Survey of Sleep Practices Among Clinicians Working With Pediatric Oncology Patients

**DOI:** 10.1002/pon.70417

**Published:** 2026-03-16

**Authors:** Lauren C. Daniel, Thembile R. Gola, Elin Irestorm, Corinne Catarozoli, Eric S. Zhou, Raphaele R. L. van Litsenburg, Valerie McLaughlin Crabtree

**Affiliations:** ^1^ Rutgers University—Department of Psychology Camden New Jersey USA; ^2^ Faculty of Medicine Department of Paediatrics Lund University Lund Sweden; ^3^ Behavioural Sciences Unit Discipline of Paediatrics & Child Health School of Clinical Medicine Randwick Clinical Campus UNSW Medicine & Health UNSW SYDNEY Kensington Australia; ^4^ Weill Cornell Medicine New York New York USA; ^5^ Department of Supportive Oncology Dana Farber Cancer Institute Boston Massachusetts USA; ^6^ Division of Sleep Medicine Harvard Medical School Boston Massachusetts USA; ^7^ Princess Máxima Center for Pediatric Oncology Utrecht the Netherlands; ^8^ Department of Psychology and Biobehavioral Sciences St. Jude Children's Research Hospital Memphis Tennessee USA

## Abstract

**Objective:**

Pediatric cancer patients are at increased risk for sleep disturbances; however, there are no clinical practice guidelines for treating sleep disturbances in pediatric oncology, resulting in variable approaches to sleep management across clinicians. The current study surveyed clinicians regarding their approaches to assessing and treating sleep.

**Methods:**

A total of 200 pediatric oncology clinicians participated in a REDCap survey about behavioral and medical sleep concerns assessed in their practice and related treatment approaches.

**Results:**

Most clinicians (61%) reported assessing sleep when patients or families raised a concern, and 10% reported never assessing sleep. Clinicians rated behavioral difficulties with sleep onset (*M* = 3.00, SD = 1.08) and insomnia (*M* = 2.91, SD = 0.99) as the most problematic sleep concerns. Sleep hygiene was the most widely endorsed intervention across almost every sleep concern. Behavioral strategies were used with similar frequency between physicians/APPs compared to other clinicians [ORs = 0.61–1.25]. Pharmacology was used more frequently by physicians/APPs than other clinicians for behavioral sleep concerns [ORs = 0.19–0.36].

**Conclusions:**

Relying on children and families to report concerns can be a missed opportunity to identify sleep disturbances early. The most endorsed treatment, sleep hygiene, is likely ineffective as a standalone treatment for complex sleep disturbances seen in oncology. It is possible that offering patients these simple but likely ineffective treatments alone may perpetuate long‐term sleep disturbances and a lack of confidence in the ability to effectively treat sleep. Further research is needed to determine the most effective treatment approaches for behavioral sleep disturbances, informing clinical practice guidelines for pediatric oncology.

## 1 Introduction

Children and adolescents receiving cancer‐directed therapy are more likely to experience sleep disturbances relative to cancer‐free peers. The myriad of potential sleep disturbances includes disease processes that influence the biological regulation of sleep [1, 2], treatment‐related side effects [3], frequent hospitalizations [4], and behavioral changes to sleep habits and patterns that impact the ability of a child to fall asleep and remain asleep throughout the night [5]. Sleep disturbances are associated with quality of life [6], symptom burden [7], fatigue [8], and mental health before and after treatment [9].

These sleep disturbances often persist into survivorship, with childhood cancer survivors reporting higher levels of insomnia, excessive daytime sleepiness, symptoms of sleep disordered breathing, and circadian rhythm sleep wake disorders than peers without cancer [10, 11, 12]. Longitudinal studies describing the prevalence of sleep disturbances across the pediatric cancer trajectory have not been conducted; however, cross‐sectional research suggests that sleep disturbances are highest during treatment [13] and the year after treatment [14], remaining elevated compared to peers and siblings into long‐term survivorship [15].

Though sleep disturbances are common, treatments tailored for pediatric cancer patients are not. Survivors report the lack of treatments for sleep disturbances as a critical unmet need, with approximately half of patients who meet criteria for a sleep disorder (42%–72% varying by sleep disorder) reporting that they wanted help, yet only 0%–5% received treatment [16]. Further, there are no clinical practice guidelines to inform the treatment of sleep disturbances during cancer treatment. This research group previously conducted a systematic review of sleep interventions in pediatric oncology, intending to use findings to inform clinical practice guidelines [17]. We identified 16 intervention studies that described a wide variety of interventions, ranging from psychoeducational, behavioral, physical activity, pharmacotherapy, and massage. Most were small pilot studies, and the synthesized findings did not support any preferred treatment. The studies were also primarily general interventions that addressed sleep as a secondary outcome. Based on these findings, the variability of treatment approaches, and the lack of research consensus to guide treatment guidelines, we surveyed clinicians to better understand current clinical approaches to sleep disturbances in pediatric oncology, for both patients on treatment and survivors. We hypothesized that clinicians would report commonly used clinical approaches in the assessment and treatment of sleep disturbances in pediatric oncology patients.

## 2 Methods

### 2.1 Participants and Procedures

Clinicians who have provided clinical care to pediatric, adolescent, and/or young adult patients with cancer over the last 5 years were eligible to participate in the current study. The study was approved by the Rutgers University IRB, and a link to an anonymous REDCap survey [18] was shared by email. Clinicians completed informed consent before starting the survey.

Clinicians were recruited through snowball recruitment methods by sharing the survey via professional networks and pediatric oncology clinician listservs. In total, 240 clinicians started the survey, 6 did not provide consent, 22 provided consent only, and 12 provided consent and demographics only. The remaining 200 records were analyzed, retaining complete (*n* = 101) and partially completed (*n* = 99) surveys in study analyses. Clinicians reported an average of 12.9 years (SD = 9.71, Range 0.3–40 years) in clinical practice. Clinician background, patient population, and world region are presented in Table 1.

**TABLE 1:** Sample demographics.

		n	%
Clinician discipline	Physicians	51	26
	Advanced practice providers (APP) (e.g., nurse practitioners, physician assistants)	38	19
	Psychologists	48	24
	Social workers	18	9
	Nurses	38	19
	Other discipline	7	3
Cancer population	Hematologic cancers	84	42
	Solid tumors	85	43
	Brain tumors	74	37
	Bone marrow/Stem cell transplant	49	25
	General oncology	67	34
	Survivorship	50	25
	Psychosocial oncology	51	26
World region	North America	164	82
	Europe	26	13
	Asia	4	2
	South America	3	1.5
	Africa	2	1
	Australia/New Zealand	1	0.5
Country income classification	High‐income country	180	90
	Low and middle‐income country	20	10

### 2.2 Measures

The survey was developed by the authorship team, including clinician‐researchers with expertise in pediatric oncology and sleep, through an iterative process (see Supplemental File 1 for survey). Survey participants were asked to provide basic demographic information about their experience and current practice in pediatric oncology and the region of the world where they practice. Clinicians were then asked to rank the problematic nature of nine behavioral (insomnia, behavioral difficulties with sleep onset, unwanted co‐sleeping, non‐restorative sleep) and medical sleep disorders (sleep disordered breathing/obstructive sleep apnea, restless legs syndrome, narcolepsy, periodic limb movement disorder, parasomnias) on a Likert scale from not problematic (0) to very problematic [5]. Insomnia and behavioral difficulties with sleep onset are highly overlapping constructs that would warrant the same International Classification of Sleep Disorders (ICSD) [19] code for diagnosis (insomnia). We included both insomnia categories due to the age range seen in pediatrics. Younger children may present with “behavioral insomnia of childhood,” when the sleep problem may be due more to caregiver limit‐setting or sleep‐onset associations (e.g., needing an external stimulus to fall asleep). On the other hand, older children may present with more classic “psychophysiological insomnia”. Clinicians were asked to identify which of the nine sleep disturbances are clinically assessed and how they approach treatment of these disturbances in their practice, including the use of non‐pharmacological approaches, pharmacological approaches, and referrals to other clinicians.

### 2.3 Data Analysis

Descriptive data were compiled to describe the sample background, frequencies of assessment strategies, and approaches to interventions. Conditions were analyzed by clinician discipline, comparing physicians and APPs to psychosocial providers using logistic regressions.

## 3 Results

### 3.1 Sleep Assessment

**FIGURE 1:**
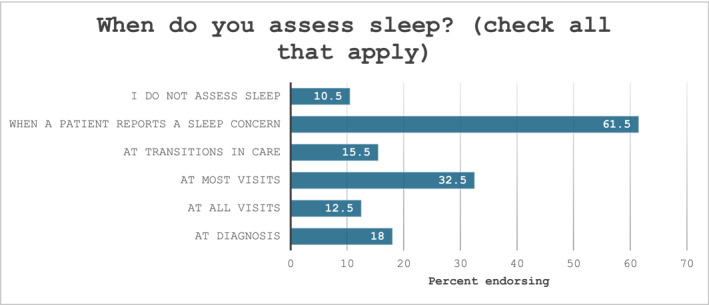
Clinician report of when sleep is assessed in oncology treatment.

Clinicians reported assessing sleep most often when a patient expressed a concern (61%; Figure 1), while 10% of respondents reported never assessing sleep. One‐third of the sample reported assessing sleep at most visits, and 15% reported assessing sleep at transitions in care. In a separate question, a small portion of the sample (*n* = 13, 6%) reported using sleep‐specific measures with patients, and the PROMIS Sleep measures were the most commonly used measures (*n* = 6).

Clinicians indicated that they typically assess three of the nine possible sleep conditions presented in this study (Median number of conditions endorsed = 3, IQR 2, 4). Twenty percent of clinicians did not endorse assessing any of the listed sleep conditions. 2% of clinicians reported assessing all nine conditions.

**FIGURE 2:**
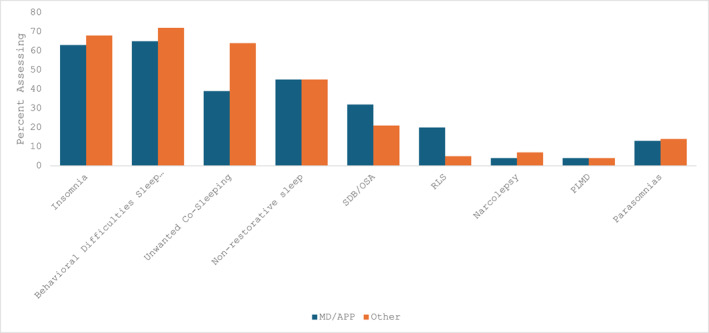
Assessment of specific sleep concerns by clinician discipline, comparing physician/advanced practice (APP) clinicians to all other clinicians (psychologists, nursing, physical therapy, occupational therapy). OSA = obstructive sleep apnea, PLMD = periodic limb movement disorder, RLS = restless legs syndrome, SDB = sleep disordered breathing.

Comparing physicians and APPs to psychosocial providers, sleep disordered breathing/obstructive sleep apnea (OR = 0.42, 95%CI 0.22, 0.79) and restless legs syndrome (OR = 0.16, 94%CI 0.06, 0.45) were assessed more frequently by physicians/APPs (Figure 2). Other sleep disturbances were assessed with similar frequency between discipline groups (Figure 2).

Clinicians rated behavioral difficulties with sleep onset (*M* = 3.00, SD = 1.08) and insomnia (*M* = 2.91, SD = 0.99) as the most problematic sleep concerns in pediatric oncology. Non‐restorative sleep (*M* = 2.88, SD = 1.12) and unwanted co‐sleeping (*M* = 2.83, SD = 1.26) were also moderately problematic. Medically based sleep disturbances, such as sleep‐disordered breathing/obstructive sleep apnea (*M* = 1.83, SD = 1.15), parasomnias (*M* = 1.35, SD = 1.07), restless legs syndrome (*M* = 1.29, SD = 1.12), narcolepsy (*M* = 0.91, SD = 1.04), and periodic limb movement disorder (*M* = 1.00, SD = 1.01) were seen as less problematic.

Most clinicians endorsed the view that sleep and fatigue are interrelated (160/172; 93%) and indicated that they treat sleep to improve cancer‐related fatigue (141/172; 82%).

### 3.2 Interventions

Clinicians endorsed insomnia, difficulties with sleep onset, unwanted co‐sleeping, and non‐restorative sleep as the most common sleep behaviors treated (Table 2). Behavioral strategies (i.e., non‐medication/supplement) were commonly used for behaviorally based sleep disturbances. Sleep hygiene was highly endorsed across sleep disturbances, excluding unwanted co‐sleeping. Sleep hygiene (118/131; 90%), relaxation (96/131; 73%), and melatonin (89/131; 68%) were the top three interventions endorsed for insomnia. Medications were used most frequently for insomnia, but they were endorsed less frequently than behavioral strategies or supplements. Referrals to mental health clinicians were common for behavioral sleep disturbances.

**TABLE 2:** Interventions for behavioral sleep conditions treated.

	Insomnia (*n* = 131)	Behavioral difficulties with sleep onset (*n* = 137)	Unwanted Co‐sleeping (*n* = 103)	Non‐restorative sleep (*n* = 90)
Non‐pharmacological				
Sleep hygiene	118	104	59	67
Cognitive behavioral therapy	52	44	32	18
Mindfulness‐based stress reduction	67	51	25	33
Relaxation	96	67	39	42
Behavioral strategies	82	90	69	30
Medications				
Antihistamines	34	19	9	13
Benzodiazepines	24	12	9	9
Hypnotics	6	6	4	2
SSRIs	13	9	3	7
Stimulants	0	1	0	2
Supplements				
Melatonin	89	69	27	34
Magnesium	18	9	4	9
Referrals				
Sleep specialist in your oncology program	12	11	9	7
Sleep specialist outside your oncology program	26	17	11	14
Mental health clinician in your oncology program	61	65	43	26
Mental health clinician outside your oncology program	19	18	18	9
Nurse in your oncology program	14	11	7	9
Other	24	11	3	6
Non‐pharmacological treatments used				
MD/APP (0)	62	57	36	36
Other clinicians (1)	65	63	51	34
Odds ratio (95% CI)	0.62 (0.34, 1.11)	0.74 (0.42, 1.31)	1.25 (0.71, 2.20)	0.65 (0.36, 1.1.67)
Pharmacological/Supplements used				
MD/APP	62	50	19	26
Other clinicians	34	24	10	13
Odds ratio (95% CI)	**0.19 (0.11, 0.35)**	**0.22 (0.12, 0.40)**	**0.37 (0.16, 0.83)**	**0.32 (0.15, 0.67)**

*Note:* Bold values denotes *p*‐value less than 0.01.

Interventions endorsed were compared by clinician type (physicians/APPs vs. others) for the behavioral treatments. There was no difference in the use of behavioral treatments by clinician type; however, all pharmacological/supplement treatments were used more frequently by physicians/APPs than by other clinician types (Table 2). Due to the smaller number of clinicians endorsing the assessment of medical sleep disturbances, we did not conduct a comparison by clinician type for treatments of medical sleep conditions.

Fewer clinicians endorsed treating medical sleep disturbances (Table 3). There were few areas of agreement on treatment approaches for medical sleep conditions, with the exception of CPAP for sleep‐disordered breathing/obstructive sleep apnea, which 26 out of 53 clinicians endorsed. Referral to a sleep specialist varied across medical conditions but was highest for sleep‐disordered breathing/obstructive sleep apnea (26/53).

**TABLE 3:** Interventions for medical sleep conditions treated.

	SDB/OSA (*n* = 53)	RLS (*n* = 25)	Narcolepsy (*n* = 11)	PLMD (*n* = 8)	Parasomnias (*n* = 27)
Non‐pharmacological					
Sleep hygiene	10	6	4	3	13
Cognitive behavioral therapy	1	3	2	0	6
Mindfulness‐based stress reduction	3	5	1	1	8
Relaxation	6	9	2	3	8
Behavioral strategies	5	2	1	3	13
Continuous positive airway pressure	26	—	—	—	—
Other	8	4	2	1	2
Medications					
Antihistamines	2	3	0	1	0
Benzodiazepines	2	4	0	0	1
Hypnotics	2	1	0	0	0
SSRIs	2	4	0	0	1
Stimulants	2	2	4	1	1
Other	3	7	4	0	1
Supplements					
Melatonin	6	3	0	1	1
Magnesium	0	8	0	1	1
Referrals					
Sleep specialist in your oncology program	8	1	3	0	3
Sleep specialist outside your oncology program	26	5	3	1	6
Mental health clinician in your oncology program	1	2	2	1	6
Mental health clinician outside your oncology program	1	1	0	0	3
Nurse in your oncology program	5	1	3	1	1
Other	9	1	4	0	1

*Note:* *Due to the small number of clinicians treating medical sleep conditions, comparisons by clinician type were not conducted.

## 4 Discussion

Approximately one‐third of pediatric oncology clinicians regularly discuss sleep with their patients, with most discussions occurring when patients raise a concern. In this survey, 10% of respondents reported not assessing sleep, suggesting the actual rate may be higher than that observed in a sample of clinicians who opted to complete a survey about sleep. Relying on children and families to report concerns can be a missed opportunity to identify sleep disturbances early, especially given the high frequency of sleep disturbances reported in pediatric oncology. Furthermore, 20% of the sample did not endorse assessing any of the nine conditions we presented, suggesting that these clinicians use a more general discussion of sleep rather than focusing on specific sleep disturbances. These results highlight the importance of proactive symptom screening that includes sleep. We are failing our patients by not identifying treatable problems. Without more information about the prevalence of sleep concerns during treatment, we limit progress toward developing effective interventions. Regular screening for sleep disturbances has been implemented in a large primary care network, resulting in an increase in sleep disorder diagnoses, orders for polysomnographies, and referrals to sleep specialists [20]. The 4‐item screener was completed electronically by families prior to the visit, and the electronic health record flagged elevated items and provided clinicians with educational materials for families. Such screening procedures are possible in oncology without overburdening families and clinicians.

The three most commonly assessed sleep disturbances were behavioral: insomnia, behavioral difficulties with sleep onset, and unwanted co‐sleeping. The assessment of behavioral and medical sleep disturbances was similar between prescribing and non‐prescribing clinicians, although prescribing clinicians were more likely to use pharmacological and/or supplement‐based treatments. Medical sleep disturbances were rarely assessed, and for clinicians who did report evaluating these symptoms, referral to sleep programs was commonly reported.

Across behavioral sleep disturbances, sleep hygiene was the most widely recommended treatment course. Sleep hygiene includes recommendations to create a sleep‐conducive environment and adopt good sleep habits to promote regular sleep, although the definition can vary widely [21]. Similarly, relaxation was broadly endorsed as the second most recommended treatment approach for insomnia. Although these recommendations are an important foundation for good sleep quality, they are insufficient as a standalone treatment for complex concerns such as insomnia [22]. These treatments typically produce only a small improvement in sleep [23] and should be combined with other behavioral and cognitive treatments that address the root causes of difficulty falling asleep and remaining asleep, such as distorted thoughts and beliefs about sleep, when seeking to treat insomnia [22]. Giving patients seemingly simple but likely ineffective steps to improve their sleep may lead to frustration with sleep as something that does not respond to treatment. This paradox of widely discussing sleep with ineffective treatments may be partially responsible for the persistent sleep concerns we have previously described in cancer survivors [15, 17, 24]. Clinicians are encouraged to discuss sleep hygiene strategies and to follow up with additional recommendations and referrals to other clinicians if sleep hygiene and relaxation do not improve sleep.

Prescribing and non‐prescribing clinicians used behavioral recommendations at similar frequencies, while prescribing clinicians were more likely to use pharmacotherapy and supplements. Hypnotics, which are not FDA‐approved in pediatrics, were rarely used. On the other hand, melatonin, antihistamines, and benzodiazepines were often used for behavioral sleep concerns. Despite the lack of evidence supporting melatonin in pediatric oncology [17], melatonin was used for insomnia more often than the gold standard cognitive behavioral therapy for insomnia (CBT‐I). A recent meta‐analysis (8 randomized controlled trials included with 419 children and adolescents) examined the evidence of melatonin as a treatment for insomnia in typically developing children [25]. The results indicated no effect for melatonin on sleep efficiency (% of time spent in bed asleep) or daytime functioning, but moderate effects, based on low‐quality evidence, for self‐reported sleep duration (increased by 30 min) and sleep onset latency (reduced by 18 min). The authors concluded that melatonin should not be the first‐line treatment for insomnia in children but may be used in the short term when other behavioral treatments have failed. Melatonin is widely available and familiar to many families in the United States, making it an acceptable treatment [26], but it is likely not addressing the root causes of sleep concerns that occur in pediatric cancer [27].

Benzodiazepines and antihistamines were used less frequently than melatonin in our survey. One study has tested diphenhydramine in children, finding small improvements in sleep parameters [sleep onset latency decreased by 8.7 min, sleep duration by 10.8 min, night awakenings reduced by 0.39 events per night [28]]. Tolerance to the sleep‐inducing effects of antihistamines develops quickly, making these medications less than ideal for chronic sleep concerns [29]. Benzodiazepines are generally only recommended for short‐term use for sleep, and because of limited safety and efficacy data in pediatrics and the potential to alter sleep architecture and induce parasomnias, these medications tend to be used less often for sleep concerns [30]. Because pediatric cancer patients often already have benzodiazepine prescriptions for other indications, use for sleep may be logical; however, clinicians are urged to consider the long‐term implications of these medications in the context of sleep, when the potential overdose may be higher [31].

Clinicians reported seeing sleep and fatigue as interrelated and often treating sleep to improve cancer‐related fatigue. A large population‐based assessment of sleep concerns in adolescent and young adult pediatric cancer survivors described that although insomnia and fatigue co‐occur, they are not synonymous (18% of the sample reported both sleep problems and fatigue, 7% fatigue only, and 13% insomnia only) [24]. Comprehensive treatment packages for sleep disturbances in adult oncology have been shown to affect some but not all facets of fatigue [32]. A recent study of CBT‐I in adolescent survivors improved both insomnia symptoms and fatigue [33]. Thus, considering comprehensive treatment packages such as CBT‐I for patients presenting with both sleep and fatigue concerns is likely the most effective approach to treating these patients. CBT‐I packages are most appropriate for older patients who can engage in cognitive therapy, and most treatment packages that have adapted CBT‐I for pediatrics have focused on adolescents [34, 35].

Other behavioral insomnia concerns that occur more often in younger children, including caregiver limit setting and sleep onset associations, are most often treated through behavioral treatments implemented with caregivers. There are solid clinical guidelines for addressing these concerns in children without cancer that can be adapted for a cancer population [36, 37], although few studies have tested these strategies to date [17]. One study piloted a multi‐modal cognitive‐behavioral intervention for children and adolescents undergoing stem cell rescue therapy for medulloblastoma. Study results indicated that the intervention helped patients maintain their longest sleep bout compared to the comparison group, who showed increasingly shorter sleep intervals, and for patients who had better nighttime sleep, next‐day fatigue was reduced [38].

Co‐sleeping during treatment is commonly assessed, though it may not always be treated. Perceptions of co‐sleeping can vary widely by family and culture; however, in this instance, we were asking the clinician's understanding of when their patients' caregivers reported co‐sleeping that was not aligned with the caregiver's parenting goals. We have previously described high rates of co‐sleeping in pediatric patients [7] and adolescent and young adult survivors [24]. That co‐sleeping remains a concern in adolescent and young adult survivors again suggests a sleep disturbance that persists beyond treatment. Co‐sleeping should likely be addressed earlier in the treatment trajectory. One case series has demonstrated that co‐sleeping can effectively be eliminated in children on active cancer‐directed therapy in preparation for stem cell transplant [39].

### 4.1 Study Limitations

The current survey provides approaches to assessing and treating sleep in children and adolescents with cancer; however, the small sample size, primarily drawn from North American institutions, is a limitation for generalizing the findings across pediatric oncology settings. The study was not powered to analyze regional differences in sleep assessment and treatment, which would be a worthy direction for a subsequent study. Clinicians who elected to complete this survey about sleep may have overestimated the rates of sleep treatment in the general population of pediatric oncology clinicians. Because our survey design allowed clinicians to select all the treatment approaches they used, we cannot determine whether clinicians used specific treatments alone or as part of a treatment package.

### 4.2 Clinical Implications

These results underscore the need to systematically assess symptoms such as sleep and fatigue in clinical practice. Following established protocols for brief sleep assessment, such as a brief screener [20], is a practical starting point. The BEARS acronym addresses the most common pediatric sleep concerns: Bedtime issues, Excessive daytime sleepiness, night Awakenings, Regularity and duration of sleep, and Snoring [40, 41]. Once behavioral sleep concerns are identified, referrals to behavioral health clinicians, ideally those familiar with cognitive behavioral therapy for insomnia, is an important step in addressing sleep concerns early. For medically based sleep disturbances such as obstructive sleep apnea and narcolepsy, referring patients for further evaluation in pediatric sleep programs is an important next step. Clinicians are also encouraged not to rely solely on sleep hygiene, relaxation, or melatonin; rather, these treatments should be considered as part of a broader cognitive‐behavioral treatment package.

## 5 Conclusions

We reiterate our prior call for the broader inclusion of sleep in pediatric oncology research [41], including more testing of interventions aimed at improving sleep [17]. Both lines of research are crucial for enhancing the health and quality of life of patients undergoing cancer treatment and for informing evidence‐based clinical practice guidelines.

## Funding

The authors have nothing to report.

## Conflicts of Interest

The authors declare no conflicts of interest.

## Supporting information


Supporting Information S1

## Data Availability

The data that support the findings of this study are available from the corresponding author upon reasonable request.
