# Supplementary material for: Survey of Sleep Practices Among Clinicians Working With Pediatric Oncology Patients
Source: Psychooncology. 2026 Mar 16;35(3):e70417. doi: 10.1002/pon.70417 (PMC12992674; doi:10.1002/pon.70417)
Supplement: Supplementary file 1 — Supporting Information S1 [file PON-35-e70417-s001.pdf]

We understand that oncology clinicians help their patients with many concerns at every visit. In this survey, we are trying to understand how you assess and treat sleep in children, adolescents, and young adults (0-21 years of age) with cancer.

We recognize that interventions for sleep problems may not always be possible given the complex medical treatment for pediatric cancer patients.

Please respond to the following questions about your clinical practice by reporting what you usually do, not what you think you should do.

Thank you!

Years in practice in pediatric oncology

____

Clinical specialty

- ○ Physician
- ○ Advanced practice provider (e.g., physician assistant, nurse practitioner)
- ○ Psychologist
- ○ Social Worker
- ○ Other

Please state clinical specialty

____

Focus within oncology (select all that apply)

- ☐ Hematologic/Liquid Malignancies
- ☐ Solid tumors
- ☐ Brain tumors
- ☐ Bone Marrow/Stem Cell Transplantation
- ☐ General oncology
- ☐ Survivorship
- ☐ Psycho-oncology

In which region of the world do you practice ?

- ○ North America
- ○ South America
- ○ Europe
- ○ Africa
- ○ Asia
- ○ Australia/New Zealand/Oceana

	High-income country	Low- and middle-income country
Please specify by World Bank Income Classification	○	○

Sleep Assessment

When do you assess sleep problems in your patients ? (Select all that apply)

- ☐ At diagnosis
- ☐ At all visits
- ☐ At most visits
- ☐ At transitions in care (i.e., changing treatment phase, before/after prolonged hospitalization)
- ☐ When a patient reports a sleep concern
- ☐ I do not assess sleep

Do you use specific measures to assess patient sleep during appointments?

- ◯ Yes
- ◯ No

What measures do you use?

____

On a scale of 0 (not problematic) to 5 (very problematic), how much of a problem are the following sleep concerns in pediatric oncology patients?

Insomnia

not problematic — somewhat problematic — very problematic

(Place a mark on the scale above)

Behavioral difficulties around falling asleep

not problematic — somewhat problematic — very problematic

(Place a mark on the scale above)

Behavioral difficulties around child sleeping alone/in own bed

not problematic — somewhat problematic — very problematic

(Place a mark on the scale above)

Non-restorative sleep

not problematic — somewhat problematic — very problematic

(Place a mark on the scale above)

Sleep disordered breathing/Obstructive sleep apnea

not problematic — somewhat problematic — very problematic

(Place a mark on the scale above)

Narcolepsy

not problematic — somewhat problematic — very problematic

(Place a mark on the scale above)

Restless leg syndrome

not problematic — somewhat problematic — very problematic

(Place a mark on the scale above)

Periodic Limb Movement Disorder

not problematic — somewhat problematic — very problematic

(Place a mark on the scale above)

Parasomnias (sleep walking/talking, enuresis, headbanging)

not problematic — somewhat problematic — very problematic

(Place a mark on the scale above)

Do you see sleep and fatigue as interrelated? ◯ Yes ◯ No

Do you address sleep to improve cancer-related fatigue?
◯ Yes
◯ No

What sleep concerns do you assess with your patients? (Select all that apply)

- ☐ Insomnia
- ☐ Behavioral difficulties around falling asleep
- ☐ Behavioral difficulties around child sleeping alone/in own bed
- ☐ Non-restorative sleep
- ☐ Sleep disordered breathing/Obstructive sleep apnea
- ☐ Restless legs syndrome
- ☐ Narcolepsy
- ☐ Periodic limb movement disorder
- ☐ Parasomnias
- ☐ Other

When seeing a patient with INSOMNIA how do you approach treatment? Select all that apply

Non-pharmacologically

- ☐ Sleep hygiene
- ☐ Cognitive behavioral therapy
- ☐ Mindfulness-based stress reduction
- ☐ Relaxation
- ☐ Behavioral sleep interventions (e.g., faded bedtime, rewards, bedtime pass, gradual withdrawal)
- ☐ Other
- ☐ None

Please specify

____

Pharmacologically

- ☐ Hypnotics (z-drugs, e.g. zolpidem, eszopiclone)
- ☐ Antihistamines
- ☐ SSRIs
- ☐ Benzodiazepines
- ☐ Stimulants
- ☐ Other
- ☐ None

Please specify

____

Supplements

- ☐ Melatonin
- ☐ Magnesium
- ☐ Other
- ☐ None

Please specify

____

Referral to

- ☐ Sleep specialist in your oncology program
- ☐ Sleep specialist outside your oncology program
- ☐ Mental health clinician in your oncology program
- ☐ Mental health clinician outside of your oncology program
- ☐ Nurse in your oncology program
- ☐ Other clinician
- ☐ None

Please specify

____

When do you address insomnia?

- ◯ When it occurs
- ◯ I do not treat this concern until cancer treatment ends
- ◯ I do not treat this concern

Of the treatments you selected, is there a treatment you typically try first for this condition?

____

When seeing a patient with BEHAVIORAL DIFFICULTIES AROUND FALLING ASLEEP how do you approach treatment? Select all that apply

Non-pharmacologically

- ☐ Sleep hygiene
- ☐ Cognitive behavioral therapy
- ☐ Mindfulness-based stress reduction
- ☐ Relaxation
- ☐ Behavioral sleep interventions (e.g., faded bedtime, rewards, bedtime pass, gradual withdrawal)
- ☐ Other
- ☐ None

Please specify

____

Pharmacologically

- ☐ Hypnotics (z-drugs, e.g. zolpidem, eszopiclone)
- ☐ Antihistamines
- ☐ SSRIs
- ☐ Benzodiazepines
- ☐ Stimulants
- ☐ Other
- ☐ None

Please specify

____

Supplements

- ☐ Melatonin
- ☐ Magnesium
- ☐ Other
- ☐ None

Please specify

____

Referral to

- ☐ Sleep specialist in your oncology program
- ☐ Sleep specialist outside your oncology program
- ☐ Mental health clinician in your oncology program
- ☐ Mental health clinician outside of your oncology program
- ☐ Nurse in your oncology program
- ☐ Other clinician
- ☐ None

Please specify

____

When do you address behavioral difficulties around falling asleep?

- ◯ When it occurs
- ◯ I do not treat this concern until cancer treatment ends
- ◯ I do not treat this concern

Of the treatments you selected, is there a treatment you typically try first for this condition?

____

When seeing a patient with BEHAVIOR DIFFICULTIES AROUND CHILD SLEEPING ALONE/IN OWN BED how do you approach treatment? Select all that apply

Non-pharmacologically

- ☐ Sleep hygiene
- ☐ Cognitive behavioral therapy
- ☐ Mindfulness-based stress reduction
- ☐ Relaxation
- ☐ Behavioral sleep interventions (e.g., faded bedtime, rewards, bedtime pass, gradual withdrawal)
- ☐ Other
- ☐ None

Please specify

____

Pharmacologically

- ☐ Hypnotics (z-drugs, e.g. zolpidem, eszopiclone)
- ☐ Antihistamines
- ☐ SSRIs
- ☐ Benzodiazepines
- ☐ Stimulants
- ☐ Other
- ☐ None

Please specify

____

Supplements

- ☐ Melatonin
- ☐ Magnesium
- ☐ Other
- ☐ None

Please specify

____

Referral to

- ☐ Sleep specialist in your oncology program
- ☐ Sleep specialist outside your oncology program
- ☐ Mental health clinician in your oncology program
- ☐ Mental health clinician outside of your oncology program
- ☐ Nurse in your oncology program
- ☐ Other clinician
- ☐ None

Please specify

____

When do you address behavioral difficulties around child sleeping alone/in own bed?

- ◯ When it occurs
- ◯ I do not treat this concern until cancer treatment ends
- ◯ I do not treat this concern

Of the treatments you selected, is there a treatment you typically try first for this condition?

____

When seeing a patient with NON-RESTORATIVE SLEEP how do you approach treatment? Select all that apply

Non-pharmacologically

- ☐ Sleep hygiene
- ☐ Cognitive behavioral therapy
- ☐ Mindfulness-based stress reduction
- ☐ Relaxation
- ☐ Behavioral sleep interventions (e.g., faded bedtime, rewards, bedtime pass, gradual withdrawal)
- ☐ Other
- ☐ None

Please specify

____

Pharmacologically

- ☐ Hypnotics (z-drugs, e.g. zolpidem, eszopiclone)
- ☐ Antihistamines
- ☐ SSRIs
- ☐ Benzodiazepines
- ☐ Stimulants
- ☐ Other
- ☐ None

Please specify

____

Supplements

- ☐ Melatonin
- ☐ Magnesium
- ☐ Other
- ☐ None

Please specify

____

Referral to

- ☐ Sleep specialist in your oncology program
- ☐ Sleep specialist outside your oncology program
- ☐ Mental health clinician in our oncology program
- ☐ Mental health clinician outside of your oncology program
- ☐ Nurse in our oncology program
- ☐ Other clinician
- ☐ None

Please specify

____

When do you address non-restorative sleep?

- ◯ When it occurs
- ◯ I do not treat this concern until cancer treatment ends
- ◯ I do not treat this concern

Of the treatments you selected, is there a treatment you typically try first for this condition?

____

When you see a patient with SLEEP DISORDERED BREATHING/OBSTRUCTIVE SLEEP APNEA how do you approach treatment? (Select all that apply)

Non-pharmacologically
- ☐ Sleep hygiene
- ☐ Cognitive behavioral therapy
- ☐ Mindfulness-based stress reduction
- ☐ Relaxation
- ☐ Behavioral sleep interventions (e.g., faded bedtime, rewards, bedtime pass, gradual withdrawal)
- ☐ Contionous positive airway pressure
- ☐ Other
- ☐ None

Please specify
____

Pharmacologically
- ☐ Hypnotics (z-drugs, e.g. zolpidem, eszopiclone)
- ☐ Antihistamines
- ☐ SSRIs
- ☐ Benzodiazepines
- ☐ Stimulants
- ☐ Other
- ☐ None

Please specify
____

Supplements
- ☐ Melatonin
- ☐ Magnesium
- ☐ Other
- ☐ None

Please specify
____

Referral to
- ☐ Sleep specialist in your oncology program
- ☐ Sleep specialist outside your oncology program
- ☐ Mental health clinician in your oncology program
- ☐ Mental health clinician outside of your oncology program
- ☐ Nurse in your oncology program
- ☐ Other clinician
- ☐ None

Please specify
____

When do you address sleep disordered breathing/obstructive sleep apnea
- ◯ When it occurs
- ◯ I do not treat this concern until cancer treatment ends
- ◯ I do not treat this concern

Of the treatments you selected, is there a treatment you typically try first for this condition?
____

When you see a patient with NARCOLEPSY how do you approach treatment? Select all that apply

Non-pharmacologically	☐ Sleep hygiene<br>☐ Cognitive behavioral therapy<br>☐ Mindfulness-based stress reduction<br>☐ Relaxation<br>☐ Behavioral sleep interventions (e.g., faded bedtime, rewards, bedtime pass, gradual withdrawal)<br>☐ Other<br>☐ None
Please specify	__________________________
Pharmacologically	☐ Hypnotics (z-drugs, e.g. zolpidem, eszopiclone)<br>☐ Antihistamines<br>☐ SSRIs<br>☐ Benzodiazepines<br>☐ Stimulants<br>☐ Other<br>☐ None
Please specify	__________________________
Supplements	☐ Melatonin<br>☐ Magnesium<br>☐ Other<br>☐ None
Please specify	__________________________
Referral to	☐ Sleep specialist in your oncology program<br>☐ Sleep specialist outside your oncology program<br>☐ Mental health clinician in our oncology program<br>☐ Mental health clinician outside of your oncology program<br>☐ Nurse in our oncology program<br>☐ Other clinician<br>☐ None
Please specify	__________________________
When do you address narcolepsy	◯ When it occurs<br>◯ I do not treat this concern until cancer treatment ends<br>◯ I do not treat this concern
Of the treatments you selected, is there a treatment you typically try first for this condition?	__________________________

When seeing a patient with RESTLESS LEG SYNDROME how do you approach treatment? Select all that apply

Non-pharmacologically

- ☐ Sleep hygiene
- ☐ Cognitive behavioral therapy
- ☐ Mindfulness-based stress reduction
- ☐ Relaxation
- ☐ Behavioral sleep interventions (e.g., faded bedtime, rewards, bedtime pass, gradual withdrawal)
- ☐ Other
- ☐ None

Please specify

____

Pharmacologically

- ☐ Hypnotics (z-drugs, e.g. zolpidem, eszopiclone)
- ☐ Antihistamines
- ☐ SSRIs
- ☐ Benzodiazepines
- ☐ Stimulants
- ☐ Other
- ☐ None

Please specify

____

Supplements

- ☐ Melatonin
- ☐ Magnesium
- ☐ Other
- ☐ None

Please specify

____

Referral to

- ☐ Sleep specialist in your oncology program
- ☐ Sleep specialist outside your oncology program
- ☐ Mental health clinician in our oncology program
- ☐ Mental health clinician outside of your oncology program
- ☐ Nurse in our oncology program
- ☐ Other clinician
- ☐ None

Please specify

____

When do you assess restless leg syndrome?

- ◯ When it occurs
- ◯ I do not treat this concern until cancer treatment ends
- ◯ I do not treat this concern

Of the treatments you selected, is there a treatment you typically try first for this condition?

____

When you see a patient with PERIODIC LIMB MOVEMENT DISORDER how do you approach treatment? Select all that apply

Non-pharmacologically	☐ Sleep hygiene<br>☐ Cognitive behavioral therapy<br>☐ Mindfulness-based stress reduction<br>☐ Relaxation<br>☐ Behavioral sleep interventions (e.g., faded bedtime, rewards, bedtime pass, gradual withdrawal)<br>☐ Other<br>☐ None
Please specify	__________________________
Pharmacologically	☐ Hypnotics (z-drugs, e.g. zolpidem, eszopiclone)<br>☐ Antihistamines<br>☐ SSRIs<br>☐ Benzodiazepines<br>☐ Stimulants<br>☐ Other<br>☐ None
Please specify	__________________________
Supplements	☐ Melatonin<br>☐ Magnesium<br>☐ Other<br>☐ None
Please specify	__________________________
Referral to	☐ Sleep specialist in your oncology program<br>☐ Sleep specialist outside your oncology program<br>☐ Mental health clinician in our oncology program<br>☐ Mental health clinician outside of your oncology program<br>☐ Nurse in our oncology program<br>☐ Other clinician<br>☐ None
Please specify	__________________________
When do you address periodic limb movement	◯ When it occurs<br>◯ I do not treat this concern until cancer treatment ends<br>◯ I do not treat this concern
Of the treatments you selected, is there a treatment you typically try first for this condition?	__________________________

When seeing a patient with PARASOMNIA how do you approach treatment? Select all that apply

Non-pharmacologically	☐ Sleep hygiene<br>☐ Cognitive behavioral therapy<br>☐ Mindfulness-based stress reduction<br>☐ Relaxation<br>☐ Behavioral sleep interventions (e.g., faded bedtime, rewards, bedtime pass, gradual withdrawal)<br>☐ Other<br>☐ None
Please specify	__________________________________
Pharmacologically	☐ Hypnotics (z-drugs, e.g. zolpidem, eszopiclone)<br>☐ Antihistamines<br>☐ SSRIs<br>☐ Benzodiazepines<br>☐ Stimulants<br>☐ Other<br>☐ None
Please specify	__________________________________
Supplements	☐ Melatonin<br>☐ Magnesium<br>☐ Other<br>☐ None
Please specify	__________________________________
Referral to	☐ Sleep specialist in your oncology program<br>☐ Sleep specialist outside your oncology program<br>☐ Mental health clinician in your oncology program<br>☐ Mental health clinician outside of your oncology program<br>☐ Nurse in your oncology program<br>☐ Other clinician<br>☐ None
Please specify	__________________________________
When do you address parasomnia	◯ When it occurs<br>◯ I do not treat this concern until cancer treatment ends<br>◯ I do not treat this concern
Of the treatments you selected, is there a treatment you typically try first for this condition?	__________________________________

Other unspecified

Are there any other sleep conditions you treat or treatment approaches you use that have not been mentioned in this survey?

◯ Yes
◯ No

Please specify

____
